# Acute kidney injury: the experience of a tertiary center of Pediatric Nephrology

**DOI:** 10.1590/2175-8239-JBN-2024-0012en

**Published:** 2024-04-29

**Authors:** Carolina Figueiredo, Ana Margarida Rocha, Liane Correia-Costa, Maria do Sameiro Faria, Teresa Costa, Conceição Mota

**Affiliations:** 1 Hospital do Divino Espírito Santo de Ponta Delgada, Serviço de Pediatria, Ilha de São Miguel, Portugal.; 2 Universidade do Porto, Instituto de Ciências Biomédicas Abel Salazar, Porto, Portugal.; 3 Centro Hospitalar Universitário de Santo António, Centro Materno-Infantil do Norte, Unidade de Nefrologia Pediátrica, Porto, Portugal.; 4 Universidade do Porto, Instituto de Saúde Pública (EPIUnit), Porto, Portugal.; 5 Universidade do Porto, Laboratório para a Investigação Integrativa e Translacional em Saúde Populacional (ITR), Porto, Portugal.; 6 Universidade do Porto e Universidade NOVA de Lisboa, Unidade de Ciências Biomoleculares Aplicadas (UCIBIO), Lisboa, Portugal.

**Keywords:** Acute Kidney Injury, Pediatrics, Etiology, Risk Factors, Treatment Outcome

## Abstract

**Introduction:**

Acute kidney injury (AKI) is an abrupt deterioration of kidney function. The incidence of pediatric AKI is increasing worldwide, both in critically and non-critically ill settings. We aimed to characterize the presentation, etiology, evolution, and outcome of AKI in pediatric patients admitted to a tertiary care center.

**Methods:**

We performed a retrospective observational single-center study of patients aged 29 days to 17 years and 365 days admitted to our Pediatric Nephrology Unit from January 2012 to December 2021, with the diagnosis of AKI. AKI severity was categorized according to Kidney Disease Improving Global Outcomes (KDIGO) criteria. The outcomes considered were death or sequelae (proteinuria, hypertension, or changes in renal function at 3 to 6 months follow-up assessments).

**Results:**

Forty-six patients with a median age of 13.0 (3.5–15.5) years were included. About half of the patients (n = 24, 52.2%) had an identifiable risk factor for the development of AKI. Thirteen patients (28.3%) were anuric, and all of those were categorized as AKI KDIGO stage 3 (p < 0.001). Almost one quarter (n = 10, 21.7%) of patients required renal replacement therapy. Approximately 60% of patients (n = 26) had at least one sequelae, with proteinuria being the most common (n = 15, 38.5%; median (P25–75) urinary protein-to-creatinine ratio 0.30 (0.27–0.44) mg/mg), followed by reduced glomerular filtration rate (GFR) (n = 11, 27.5%; median (P25–75) GFR 75 (62–83) mL/min/1.73 m^2^).

**Conclusions:**

Pediatric AKI is associated with substantial morbidity, with potential for proteinuria development and renal function impairment and a relevant impact on long-term prognosis.

## Introduction

Acute kidney injury (AKI) is an abrupt deterioration of kidney function^1,2,3^. The spectrum of manifestations is wide, ranging from subtle analytic changes in renal function to symptomatic organ failure^4,5^. According to the literature, AKI affects almost one-third of hospitalized children, and its incidence is increasing worldwide^6,7,8,9,10 11^. Within a non-critically ill setting, a recent study carried out at a tertiary care children’s hospital with over two thousand patients described that AKI was observed in at least 5% of patients^4,10,12^. The frequency of AKI is particularly elevated in critically ill patients, as it is stated as the most common complication in children admitted to a pediatric intensive care unit (PICU)^13–15^. A multinational prospective study involving almost five thousand children and young adults aged 3 months to 25 years admitted to a PICU reported an incidence of AKI of 26.9%^16^. However, the overall incidence of AKI within the pediatric population is somewhat uncertain, since it depends on the population studied. A relevant body of research has focused on high-risk patients, particularly those who have been exposed to nephrotoxins, have undergone cardiac surgery, or have been admitted to a PICU^4,12^.

Multiple pathophysiological mechanisms might be involved in AKI. Pre-renal etiologies are currently the most commonly associated with pediatric AKI, followed by intrinsic or renal disorders, such as glomerulonephritis^14,17,18^. Since few effective specific therapeutic approaches are available today, knowledge of the risk factors for AKI is of paramount importance^1,19,20^. Factors like prematurity or chronic comorbidities and events such as volume depletion, nephrotoxin exposure, sepsis, and major surgery (cardiac surgery, mainly with cardiopulmonary bypass) are the preponderant factors for the development of AKI^1,2,5^.

Concerning short-term outcomes, several studies have concluded that AKI in hospitalized pediatric patients may lead to prolonged mechanical ventilation, longer length of stay, and greater mortality^3,7,18,21^. Also, AKI may be associated with later development of proteinuria, hypertension, and chronic kidney disease^3,7,9,18,19,22,23,24^.

In the present study, we aimed to characterize the presentation, etiology, evolution, and outcome of all cases of AKI in pediatric patients aged 29 days to 17 years and 365 days admitted to a tertiary of pediatric nephrology center in Portugal in the last decade.

## Methods

### Study Design and Sample

We conducted a retrospective observational single-center cohort study of children and adolescents aged 29 days to 17 years and 365 days admitted to the Nephrology Unit of Centro Materno-Infantil do Norte for a period of 10 consecutive years (from January 2012 to December 2021) with the diagnosis of AKI. All patients with AKI diagnosis at discharge were included, unless there was a previous diagnosis of chronic kidney disease (16 patients were excluded from the present analysis since stages 2–4 chronic kidney disease was present and the observed injury was considered an acute-on-chronic kidney injury).

### Data Collection and Variables Definition

Clinical data were retrieved from the electronic clinical records of the included patients.

AKI severity was assessed using the Kidney Disease Improving Global Outcomes (KDIGO) stages 1–3, which were defined based on the baseline and maximum inpatient serum creatinine (SCr) values recorded, as follows: stage 1 AKI was defined as a SCr value of 1.5 to 1.9 times the baseline value or ≥0.3 mg/dL increase, or urine volume <0.5 mL/kg/h for 6 to 12 hours; stage 2 AKI was defined as a SCr value of 2.0 to 2.9 times the baseline value or urine volume <0.5 mL/kg/h for ≥12 hours; stage 3 AKI comprised a SCr value 3.0 times the baseline value or increase in SCr to ≥4.0 mg/dL or the initiation of renal replacement therapy or decrease in estimate glomerular filtration rate (GFR) to <35 mL/min per 1.73 m^2^, or urinary volume <0.3 mL/kg/h for ≥24 hours, or anuria for ≥12 hours^2^.

The baseline SCr value was considered to be the lowest value within 6 months prior to admission (including the value at admission); all creatinine measurements were performed by the enzymatic method. GFR was calculated based on the revised Schwartz formula, k×(height(cm)/SCr(mg/dL)); using a k constant of 0.413.

Proteinuria was defined as a urinary ratio of protein/creatinine (uP/C) >0.2 mg/mg. Hematuria was defined as ≥5 red blood cells per high-power field in urine microscopy analysis.

Both in-hospital and office BP measurements were evaluated with oscillometric validated sphygmomanometers with an adequately sized cuff in the right arm, with the child in a seated position and the antecubital fossa supported at heart level, at least twice (ideally three times), with a 1-minute interval between measurements. The last available value was considered for analysis. Age-, sex-, and height-specific SBP and DBP reference values were considered for BP classification, according to the reference values of the European Hypertension Society guidelines (hypertension if the systolic or diastolic values were at or above the 95^th^ percentile)^25^.

The need for renal biopsy and kidney replacement therapy was recorded in all patients. Data on admission to the intensive care unit, including the need for mechanical ventilation and the use of inotropes, was recorded.

The diagnosis of acute interstitial nephritis was based on clinical criteria in all patients, but in 4 cases a kidney biopsy was performed. The following risk factors were considered: comorbidities, which included previous kidney, cardiovascular, hemato-oncologic, or autoimmune diseases; exposure to nephrotoxins; prematurity; the presence of congenital anomalies of the kidney and urinary tract (CAKUT); and nephrolithiasis.

The outcomes considered were sequelae and death. Sequelae were defined as the presence of at least one of the following: proteinuria, hypertension, or reduced GFR, defined as GFR <90 mL/min/1.73 m^2^, based on clinical and analytical monitoring 3 to 6 months after discharge.

### Ethics

The project “Acute kidney injury - the experience of a tertiary center of Pediatric Nephrology” was approved by the Department of Education and Research and by the Ethical Commission of Centro Hospitalar Universitário do Porto. It complies with the Helsinki Declaration, the guidelines for the ethical conduct of medical research involving children, and the current national legislation.

### Statistical Analysis

Standard statistical analysis was performed using IBM SPSS Statistics for Macintosh, Version 28.0.1.0 (Armonk, NY: IBM Corp, USA). The variables are presented as median and 25^th^ and 75^th^ percentiles or n (%), as appropriate. Differences between groups for continuous variables were evaluated with Mann-Whitney test. Chi-square test was used for the comparison of proportions of categorical variables. A p-value of less than 0.05 was considered significant.

## Results

A total of 46 pediatric patients with a median (25^th^–75^th^ percentile, P25–75) age of 13.0 (3.5–15.5) years were included in the analysis. Demographic characteristics of both clinical and analytic parameters are shown in Table 1, according to the KDIGO stage [stage 1, 10 (21.7%); stage 2, 12 (26.1%); stage 3, 24 (52.2%)]. The main pathogenic mechanism reported was intrinsic renal causes (73.9%). The most common etiologies of AKI were acute interstitial nephritis (23.9%), dehydration/shock (21.7%), and acute glomerulonephritis (19.6%). Approximately half of the patients (n = 24, 52.2%) had an identifiable risk factor for the development of AKI, most common comorbidities (37.5%) were pathologies (renal: 1 nephrotic syndrome and 1 hematoproteinuria under investigation; cardiovascular: 1 hypertension, 1 ventricular septal defect, and 1 internal jugular vein thrombosis; hemato-oncologic: 1 osteoid osteoma; autoimmune: 1 type 1 diabetes and systemic lupus erythematosus and 1 autoimmune hepatitis), followed by exposure to nephrotoxins (25.0%), and the presence of a CAKUT (25.0%). In regard to the classification of AKI in terms of urinary output, 13 patients (28.3%) were anuric, 7 (15.2%) were oliguric, and 26 (56.5%) were non-oliguric. All anuric patients were categorized as KDIGO stage 3 AKI. Nine (19.5%) patients presented fluid overload and all of those were classified as stage 3 AKI (considering creatinine values corrected for fluid overload). The proportion of patients with hyponatremia [KDIGO stage 1 vs stage 2 vs stage 3: 0 (0.0%) vs 1 (8.3%) vs 8 (33.3%), respectively, p = 0.043]; hyperkalemia [KDIGO stage 1 vs stage 2 vs stage 3: 0 (0.0%) vs 1 (8.3%) vs 8 (33.3%), respectively, p = 0.043] and metabolic acidosis [KDIGO stage 1 vs stage 2 vs stage 3: 1 (10.0%) vs 0 (0.0%) vs 10 (41.7%), respectively, p = 0.011] increased across AKI stages. The proportion of patients with hypertension was higher among stage 3 AKI patients but the difference was not statistically significant. Renal biopsy was performed in 10 patients, 5 of whom had stage 3 AKI (4 acute interstitial nephritis). Almost one quarter (n = 10, 21.7%) of patients required kidney replacement therapy, namely peritoneal dialysis, hemodialysis, or both techniques, all of them from the stage 3 AKI group. Ten (21.7%) patients were admitted in an intensive care unit, 90% of which had stage 3 AKI.

**Table 1:** Clinical and analytical parameters according to AKI KDIGO stages

	Total 46	KDIGO stage			*p*
		1 10 (21.7%)	2 12 (26.1%)	3 24 (52,2%)	
**Demography and anthropometry**					
Age (years)	12.96 (3.54–15.54)	13.50 (9.17–15.52)	14.21 (7.15–16.98)	10.54 (1.40–15.08)	0.109
Male sex	24 (52.2%)	3 (30.0%)	7 (58.3%)	14 (58.3%)	0.284
**AKI pathogenesis and diagnosis**					
*Prerenal*	9 (19.6%)	0 (0.0%)	2 (16.7%)	7 (29.2%)	0.122
Dehydration/shock	10 (21.7%)	1 (10.0%)	2 (16.7%)	7 (29.2%)	0.116
*Renal*	34 (73.9%)	8 (80.0%)	10 (83.3%)	16 (66.7%)	0.122
Acute interstitial nephritis	11 (23.9%)	2 (20.0%)	3 (25.0%)	6 (25.0%)	0.116
Acute glomerulonephritis	9 (19.6%)	2 (20.0%)	5 (41.7%)	2 (8.3%)	0.116
Hemolytic uremic syndrome	6 (13.0%)	0 (0.0%)	0 (0.0%)	6 (25.0%)	0.116
Nephrotoxicity	4 (8.7%)	2 (20.0%)	1 (8.3%)	1 (4.2%)	0.116
Nephrotic syndrome	1 (2.2%)	0 (0.0%)	0 (0.0%)	1 (4.2%)	0.116
*Postrenal*	3 (6.5%)	2 (20%)	0 (0.0%)	1 (4.2%)	0.122
Obstruction	3 (6.5%)	2 (20.0%)	0 (0.0%)	1 (4.2%)	0.116
Unknown	2 (4.3%)	1 (10.0%)	1 (8.3%)	0 (0.0%)	0.116
**Risk factor**	24 (52.2%)	6 (60.0%)	7 (58.3%)	11 (45.8%)	0.665
Comorbidities	9 (37.5%)	1 (16.7%)	3 (42.9%)	5 (45.5%)	0.409
Nephrotoxic	6 (25.0%)	2 (33.3%)	3 (42.9%)	1 (9.1%)	0.409
Prematurity	1 (4.2%)	1 (16.7%)	0 (0.0%)	0 (0.0%)	0.409
CAKUT	6 (25.0%)	1 (16.7%)	1 (14.3%)	4 (36.4%)	0.409
Nephrolithiasis	2 (8.3%)	1 (16.7%)	0 (0.0%)	1 (9.1%)	0.409
**Urinary Output**					
Anuria	13 (28.3%)	0 (0.0%)	0 (0.0%)	13 (54.2%)	**<0.001**
Oliguria	7 (15.2%)	1 (10.0%)	5 (41.7%)	1 (4.2%)	**<0.001**
Non-oliguria	26 (56.5%)	9 (90.0%)	7 (58.3%)	10 (41.7%)	**<0.001**
**Hypertension**	17 (37.0%)	3 (30.0%)	3 (25.0%)	11 (45.8%)	0.416
**Biochemical parameters**					
Hyponatremia	9 (19.6%)	0 (0.0%)	1 (8.3%)	8 (33.3%)	**0.043**
Hyperkalemia	9 (19.6%)	0 (0.0%)	1 (8.3%)	8 (33.3%)	**0.043**
Metabolic acidosis	11 (23.9%)	1 (10.0%)	0 (0.0%)	10 (41.7%)	**0.011**
Proteinuria	29 (63.0%)	6 (60.0%)	7 (58.3%)	16 (66.7%)	0.865
Hematuria	20 (43.5%)	6 (60.0%)	5 (41.7%)	9 (37.5%)	0.478
**Renal biopsy**	10 (21.7%)	1 (10.0%)	4 (33.3%)	5 (20.8%)	0.413
**Kidney replacement therapy**	10 (21.7%)	0 (0.0%)	0 (0.0%)	10 (41.7%)	**0.003**
Hemodialysis	2 (4.3%)	0 (0.0%)	0 (0.0%)	2 (8.3%)	
Peritoneal dialysis	6 (13.0%)	0 (0.0%)	0 (0.0%)	6 (25.0%)	
Both	2 (4.3%)	0 (0.0%)	0 (0.0%)	2 (8.3%)	
**Need for intensive care unit**	10 (21.7%)	1 (10.0%)	0 (0.0%)	9 (37.5%)	**0.022**
Mechanical ventilation	3 (6.5%)	0 (0.0%)	0 (0.0%)	3 (6.5%)	0.230
Ionotropics	2 (4.3%)	0 (0.0%)	0 (0.0%)	2 (8.3%)	0.384

AKI, acute kidney injury CAKUT, congenital anomalies of the kidney and urinary tract.

The values are reported as median (percentile 25^th^-percentile 75^th^) or n (%).

The sequela characterization according to AKI stages is shown in Table 2. Of the original 46 patients, 2 were lost to follow-up and consequently were not included in the analysis. No deaths occurred. The majority of patients (n = 26, 59.1%) had at least one sequela 3–6 months after discharge. The frequency of sequela increased across AKI KDIGO stages. The most frequent sequelae were proteinuria (n = 15, 38.5%; median (P25–75) uP/C 0.30 (0.27–0.44) mg/mg), followed by reduced GFR (n = 11, 27.5%; median (P25–75) GFR 75 (62–83) mL/min/1.73 m^2^) and hypertension (n = 4, 9.1%). Among the patients without any sequelae at follow-up, the median values of uP/C and GFR were 0.10 (0.06–0.15) mg/mg and 108 (100–119) mL/min/1.73 m^2^, respectively. Although within the normal range, the median GFR at follow-up increased across AKI stages [KDIGO stage 1 vs stage 2 vs stage 3: 93 (93-93) vs 107 (103–115) vs 115 (106–137) mL/min/1.73 m^2^, respectively, p = 0.035]. Twelve of the 15 patients with proteinuria were started on angiotensin-converting enzyme inhibitor or angiotensin II receptor blocker therapy during follow-up.

**Table 2:** Sequela characterization according to AKI KDIGO stages

	Total n = 44^a^	KDIGO stage			*p*
		1	2	3	
**Without any sequelae**	**18 (40.9%)**	**6 (60.0%)**	**6 (54.5%)**	**6 (26.1%)**	0.108
uP/C (mg/mg)	0.10 (0.06–0.15)	0.09 (0.06–0.15)	0.11 (0.07–0.16)	0.11 (0.06–0.15)	0.778
GFR (mL/min/1.73 m^2^)	108 (100–119)	93 (93-93)	107 (103–115)	115 (106–137)	**0.035**
**With sequelae**	**26 (59.1%)**	**4 (40.0%)**	**5 (45.5%)**	**17 (73.9%)**	0.108
**Proteinuria^b^**	15 (38.5%)	2 (22.2%)	2 (18.2%)	11 (57.9%)	0.051
uP/C (mg/mg)	0.30 (0.27–0.44)	(0.29–0.36)^c^	(0.23–0.29)^c^	0.30 (0.27–0.47)	0.792
**Reduced GFR^d^**	11 (27.5%)	2 (28.6%)	2 (18.2%)	7 (31.8%)	0.709
GFR (mL/min/1.73 m^2^)	75 (62-83)	(71–77)^c^	(75–79)^c^	72 (59–80)	0.633
**Hypertension^e^**	4 (9.1%)	0 (0.0%)	2 (18.2%)	2 (8.7%)	0.349

KDIGO, Kidney Disease Improving Global Outcomes GFR, glomerular filtration rate; uP/C, Urinary Protein to Creatinine Ratio.

The values are reported as n (%) and median (25^th^–75^th^ percentile).

^a^From the initial 46 patients, 2 were lost to follow-up and consequently were not included in the analysis.

^b^Proteinuria was defined as a urinary ratio of proteinuria/creatinine >0.2 mg/mg.

^c^Due to the low number of patients in this category (less than 3), median (25^th^–75^th^ percentiles) values could not be calculated and the actual values of each patient is presented.

^d^Reduced GFR was defined as GFR < 90 mL/min/1.73 m^2^.

^e^Hypertension was defined as systolic or diastolic values above the 95^th^ percentile for age, sex, and height^25^.

## Discussion

In the present study, we report the etiology, severity, and outcomes of AKI among patients admitted to a pediatric Nephrology Unit at a tertiary care hospital in the last decade.

Most of AKI cases were associated with intrinsic renal causes, especially acute interstitial nephritis, acute glomerulonephritis, and hemolytic uremic syndrome, followed by prerenal causes, namely dehydration/shock. Although several studies report that pediatric AKI is mainly derived from pre-renal etiologies^14,17,18^, the predominance of renal causes might be related to the highly differentiated nature of our center. Since we are the reference center for pediatric patients with kidney diseases for the entire northern region of the country, the proportion of renal etiologies might be overrepresented in our sample.

Patients with comorbidities are known to be highly susceptible to AKI^1,2,5^. Nephrotic syndrome, for instance, is a frequent cause of kidney disease in children, and AKI is described as a potential complication^26,27^. Although the incidence of AKI in children with nephrotic syndrome is variable among studies, a study found AKI in about half of its population^27^. In our study, though, only one patient presented AKI with a nephrotic syndrome relapse. Cardiovascular diseases, such as heart failure and congenital heart disease, also impose a significant risk for AKI^28^. AKI is particularly common in children undergoing cardiac surgery, with studies suggesting a significant correlation between moderate to severe forms of injury and postoperative mortality^29^, which is in line with our findings. AKI is a common comorbidity of hemato-oncologic diseases, and reports indicate that, in these patients, stages 2 and 3 AKI are associated with greater mortality^30^. Autoimmune diseases may also lead to the development of AKI, having the potential for rapid progression to severe forms of injury^31^. In our cohort, about a third of the patients had a previous history of either kidney, cardiovascular, hemato-oncologic, or autoimmune diseases. It is also of notice that most of these patients developed moderate to severe AKI, corresponding to KDIGO stages 2 and 3, therefore consistent with previous reports.

Oligoanuria was reported in approximately half of the admitted patients, with almost all of these patients developing stages 2 and 3 AKI, suggesting that these changes in the urinary output might represent a risk for more severe forms of disease and therefore potentially worse outcomes, as previously reported in the literature^32^.

Patients with more severe AKI were those with more biochemical parameters disturbances, such as hyponatremia, hyperkalemia, and metabolic acidosis. These findings suggest that severe renal insults are associated with more pronounced hydro-electrolytic disorders and are consistent with studies that suggest an association between electrolyte abnormalities, mainly metabolic acidosis, and worse prognosis in children with AKI^33,34^.

We reported that all patients requiring kidney replacement therapy were categorized in the most severe AKI stage, which is in agreement with previous studies^18,35^. Peritoneal dialysis was the most commonly used kidney replacement therapy, which is consistent with several studies reporting that peritoneal dialysis is a well-tolerated method, easy to perform, and with known effectiveness in the context of pediatric AKI^36,37,38^. Also, hemodialysis requires a well-functioning vascular access and hemodynamically stable patients, and is therefore reserved for more specific settings^38^. Although continuous renal replacement therapies tend to be the modality of choice in critically ill and hemodynamically unstable patients^39,40^, peritoneal dialysis was the most common kidney replacement therapy used for the patients in PICU, and no continuous therapies was used in our population within the study period.

In our study, we found that most patients who required kidney replacement therapy were also admitted to the PICU during the course of the hospitalization, highlighting the severity inherent to stage 3 AKI. Although previous studies have found a correlation between AKI severity and the need for and duration of mechanical ventilation^16,41^, we did not find a statistically significant difference in the need for mechanical ventilation at different AKI stages. This may be due to the small number of patients within our population that required PICU treatment and mechanical ventilation. The low utilization rates of mechanical ventilation and vasoactive drugs in our study cohort might suggest a lower severity of cases compared to other series, and might contribute to the absence of deaths in our cohort.

Although there were no deaths in our study, we highlight the fact that almost 60% of the patients had at least one sequela 3 to 6 months after hospital discharge, with more than 25% showing reduced GFR at follow-up, thus not completely recovering normal renal function. The finding of increasing median values of GFR at the follow-up visit across AKI stages, with higher values among patients with more severe AKI seems counterintuitive but can represent an initial stage of hyperfiltration in patients with more severe nephron loss during the AKI episode, as previously reported in the literature^42,43,44^.

We acknowledge that our study had some limitations, particularly the retrospective design and the experience of a single tertiary care center. Despite these limitations, we believe we have described a fairly representative population of pediatric AKI patients from the northern region of our country over a long period of time. We believe that the presented study contributes to increase the knowledge on AKI epidemiology, an area in need of more studies to raise awareness on the long-term consequences of AKI in pediatrics.

In conclusion, AKI was common in the pediatric setting, mainly in patients with previous comorbidities, but also affected children without a known risk factor, emphasizing the importance of early suspicion of this condition. We also found that higher severity of AKI was associated with electrolyte disturbances, the need for kidney replacement therapies, and admission to the PICU. Our results suggest AKI may be associated with significant morbidity, particularly the development of proteinuria and a reduction in GFR, and therefore renal function impairment. This highlights the need for more studies focusing on the long-term impact of AKI in order to better understand the potential for transient or permanent consequences, with important impact in the long-term follow-up and management of these patients.

## Notes

This document has an erratum: https://doi.org/10.1590/2175-8239-JBN-2024-0012eren
