# Erratum: Acute kidney injury: the experience of a tertiary center of Pediatric Nephrology

**DOI:** 10.1590/2175-8239-JBN-2024-0012eren

**Published:** 2024-07-26

In the article “Acute kidney injury: the experience of a tertiary center of Pediatric Nephrology”, with DOI code number https://doi.org/10.1590/2175-8239-JBN-2024-0012en, published in the Brazilian Journal of Nephrology, 46(3):e20240012, 2024:

**Where it was written:**

Carolina Silva Figueiredo^1^

Ana Margarida Neto da Rocha^2^

Liane Maria Correia Rodrigues da Costa Nogueira Silva^2,3,4,5^

Maria do Sameiro Pinto César de Faria^3,6^

Teresa Maria Tavares Vieira da Costa Tavares^3^

Maria da Conceição Oliveira Costa Mota^3^

**Should read:**

Carolina Figueiredo^1^

Ana Margarida Rocha^2^

Liane Correia-Costa^2,3,4,5^

Maria do Sameiro Faria^3,6^

Teresa Costa^3^

Conceição Mota^3^

