# Supplementary material for: Acute kidney injury: the experience of a tertiary center of Pediatric Nephrology
Source: J Bras Nefrol. 2024 Apr 29;46(3):e20240012. doi: 10.1590/2175-8239-JBN-2024-0012en (PMC11299983; doi:10.1590/2175-8239-JBN-2024-0012en)
Supplement: Translation

# Translation: Insuficiência renal aguda: experiência de um centro terciário de Nefrologia Pediátrica

**DOI:** 10.1590/2175-8239-JBN-2024-0012pt

**Authors:** Carolina Figueiredo, Ana Margarida Rocha, Liane Correia-Costa, Maria do Sameiro Faria, Teresa Costa, Conceição Mota

**Affiliations:** 1 Hospital do Divino Espírito Santo de Ponta Delgada, Serviço de Pediatria, Ilha de São Miguel, Portugal.; 2 Universidade do Porto, Instituto de Ciências Biomédicas Abel Salazar, Porto, Portugal.; 3 Centro Hospitalar Universitário de Santo António, Centro Materno-Infantil do Norte, Unidade de Nefrologia Pediátrica, Porto, Portugal.; 4 Universidade do Porto, Instituto de Saúde Pública (EPIUnit), Porto, Portugal.; 5 Universidade do Porto, Laboratório para a Investigação Integrativa e Translacional em Saúde Populacional (ITR), Porto, Portugal.; 6 Universidade do Porto e Universidade NOVA de Lisboa, Unidade de Ciências Biomoleculares Aplicadas (UCIBIO), Lisboa, Portugal.

**Keywords:** Injúria Renal Aguda, Pediatria, Etiologia, Fatores de Risco, Resultado do Tratamento

## Abstract

**Introdução:**

Insuficiência renal aguda (IRA) é uma deterioração abrupta da função renal. A incidência de IRA pediátrica está aumentando em todo o mundo, em ambientes críticos e não críticos. Nosso objetivo foi caracterizar apresentação, etiologia, evolução e desfechos da IRA em pacientes pediátricos internados em um centro de atendimento terciário.

**Métodos:**

Realizamos estudo retrospectivo observacional de centro único de pacientes com idade entre 29 dias a 17 anos e 365 dias internados em nossa Unidade de Nefrologia Pediátrica, de janeiro de 2012 a dezembro de 2021, com diagnóstico de IRA. A gravidade da IRA foi categorizada de acordo com os critérios do **Kidney Disease Improving Global Outcomes** (KDIGO). Os desfechos considerados foram óbito ou sequelas (proteinúria, hipertensão ou alterações na função renal em avaliações de acompanhamento de 3 a 6 meses).

**Resultados:**

Incluímos 46 pacientes com idade mediana de 13,0 (3,5–15,5) anos. Cerca de metade (n = 24; 52,2%) apresentou um fator de risco identificável para o desenvolvimento de IRA. Treze pacientes (28,3%) eram anúricos; todos foram classificados como IRA KDIGO 3 (p < 0,001). Quase um quarto (n = 10; 21,7%) dos pacientes necessitaram de terapia renal substitutiva. Aproximadamente 60% (n = 26) apresentou pelo menos uma sequela, sendo proteinúria a mais comum (n = 15; 38,5%; mediana (P25–75) da relação proteína/creatinina urinária 0,30 (0,27–0,44) mg/mg), seguida de taxa de filtração glomerular (TFG) reduzida (n = 11; 27,5%; mediana (P25–75) da TFG 75 (62–83) mL/min/1,73 m^2^).

**Conclusões:**

A IRA pediátrica está associada à morbidade substancial, com potencial para desenvolvimento de proteinúria e comprometimento da função renal e impacto relevante no prognóstico de longo prazo.

## Introdução

A insuficiência renal aguda (IRA) é uma deterioração abrupta da função renal^1–3^. O espectro de manifestações é amplo, variando de alterações analíticas sutis na função renal à falência do órgão^4,5^. De acordo com a literatura, a IRA afeta quase um terço de crianças hospitalizadas, e sua incidência está aumentando em todo o mundo^6–11^. Em ambiente não crítico, um estudo recente realizado em um hospital infantil de cuidados terciários com mais de dois mil pacientes descreveu que a IRA foi observada em pelo menos 5% deles^4,10,12^. A frequência de IRA é particularmente elevada em pacientes críticos, pois a mesma é considerada a complicação mais comum em crianças internadas em unidades de terapia intensiva pediátrica (UTIP)^13–15^. Um estudo prospectivo multinacional envolvendo quase cinco mil crianças e adultos jovens com idades entre 3 meses e 25 anos internados em UTIP relatou uma incidência de IRA de 26,9%^16^. No entanto, a incidência geral de IRA na população pediátrica é um pouco incerta, uma vez que depende da população estudada. Um corpo relevante de pesquisas tem se concentrado em pacientes de alto risco, especialmente aqueles que foram expostos a nefrotoxinas, submetidos a cirurgia cardíaca ou internados em UTIP^4,12^.

Diversos mecanismos fisiopatológicos podem estar envolvidos na IRA. Atualmente, as etiologias pré-renais são as mais comumente associadas à IRA pediátrica, seguidas por distúrbios intrínsecos ou renais, como a glomerulonefrite^14,17,18^. Uma vez que poucas abordagens terapêuticas específicas e eficazes estão disponíveis no momento, o conhecimento dos fatores de risco para IRA é de suma importância^1,19,20^. Fatores como prematuridade ou comorbidades crônicas e eventos como depleção de volume, exposição a nefrotoxinas, sepse e cirurgias de grande porte (cirurgias cardíacas, principalmente com circulação extracorpórea) são os fatores preponderantes para o desenvolvimento de IRA^1,2,5^.

Com relação aos desfechos de curto prazo, diversos estudos têm concluído que a IRA em pacientes pediátricos hospitalizados pode levar à ventilação mecânica prolongada, maior tempo de internação e maior mortalidade^3,7,18,21^. Além disso, a IRA pode estar associada ao desenvolvimento posterior de proteinúria, hipertensão e doença renal crônica^3,7,9,18,19,22–24^.

No presente estudo, nosso objetivo foi caracterizar a apresentação, etiologia, evolução e desfechos de todos os casos de IRA em pacientes pediátricos com idade entre 29 dias e 17 anos e 365 dias internados em um centro terciário de nefrologia pediátrica em Portugal na última década.

## Métodos

### Desenho do Estudo e Amostra

Realizamos um estudo de coorte retrospectivo observacional de centro único de crianças e adolescentes com idades entre 29 dias e 17 anos e 365 dias internados na Unidade de Nefrologia do Centro Materno-Infantil do Norte por um período de 10 anos consecutivos (de janeiro de 2012 a dezembro de 2021) com o diagnóstico de IRA. Foram incluídos todos os pacientes com diagnóstico de IRA no momento da alta hospitalar, a menos que houvesse um diagnóstico prévio de doença renal crônica (16 pacientes foram excluídos da presente análise, uma vez que apresentavam doença renal crônica nos estágios 2 a 4 e a lesão observada foi considerada uma lesão renal aguda sobreposta à doença renal crônica).

### Coleta de Dados e Definição de Variáveis

Os dados clínicos foram recuperados de prontuários clínicos eletrônicos dos pacientes incluídos.

A gravidade da IRA foi avaliada usando os estágios 1 a 3 do *Kidney Disease Improving Global Outcomes* (KDIGO), que foram definidos com base nos valores de creatinina sérica basal e máximo registrados durante a internação, da seguinte forma: IRA estágio 1 foi definido como um valor de creatinina sérica (CrS) de 1,5 a 1,9 vezes o valor basal, ou aumento de ≥0,3 mg/dL, ou volume urinário <0,5 mL/kg/h por 6 a 12 horas; IRA estágio 2 foi definido como um valor de CrS de 2,0 a 2,9 vezes o valor basal, ou volume urinário <0,5 mL/kg/h por ≥12 horas; IRA estágio 3 compreendia um valor de CrS 3,0 vezes o valor basal, ou aumento na CrS para ≥4,0 mg/dL, ou início de terapia renal substitutiva, ou diminuiçãoda taxa de filtração glomerular (TFG) estimada para <35 mL/min por 1,73 m^2^, ou volume urinário <0,3 mL/kg/h por ≥24 horas, ou anúria por ≥12 horas^2^.

O valor basal de CrS foi considerado o valor mais baixo nos 6 meses anteriores à internação (incluindo o valor no momento da internação); todas as medições de creatinina foram realizadas pelo método enzimático. A TFG foi calculada com base na fórmula revisada de Schwartz, k×(altura(cm)/creatinina sérica(mg/dL)); usando uma constante k de 0,413.

A proteinúria foi definida como uma relação proteína/creatinina urinária (PU/CU) >0,2 mg/mg. A hematúria foi definida como ≥5 eritrócitos por campo de grande aumento na análise de microscopia da urina.

As medidas de PA no hospital e no consultório foram avaliadas com esfigmomanômetros oscilométricos validados utilizando um manguito de tamanho adequado no braço direito, com a criança em posição sentada e a fossa antecubital apoiada no nível do coração, pelo menos duas vezes (idealmente três vezes), com um intervalo de 1 minuto entre as medições. O último valor disponível foi considerado para análise. Para a classificação da PA, foram considerados valores de referência de PAS e PAD específicos para idade, sexo e altura, de acordo com os valores de referência das diretrizes da Sociedade Europeia de Hipertensão (hipertensão se os valores sistólicos ou diastólicos estivessem dentro ou acima do percentil 95)^25^.

Em todos os pacientes, foi registrada a necessidade de biópsia renal e terapia renal substitutiva. Foram registrados dados sobre a admissão na unidade de terapia intensiva, incluindo a necessidade de ventilação mecânica e o uso de inotrópicos.

O diagnóstico de nefrite intersticial aguda foi baseado em critérios clínicos em todos os pacientes, mas em quatro casos foi realizada uma biópsia renal. Foram considerados os seguintes fatores de risco: comorbidades, que incluíram doenças renais, cardiovasculares, hemato-oncológicas ou autoimunes anteriores; exposição a nefrotoxinas; prematuridade; presença de anomalias congênitas do rim e do trato urinário (CAKUT); e nefrolitíase.

Os desfechos considerados foram sequelas e óbito. As sequelas foram definidas como a presença de pelo menos um dos eventos a seguir: proteinúria, hipertensão ou TFG reduzida, definida como TFG < 90 mL/min/1,73 m^2^, com base no monitoramento clínico e analítico de 3 a 6 meses após a alta.

### Ética

O projeto “Insuficiência renal aguda - experiência de um centro terciário de Nefrologia Pediátrica” foi aprovado pelo Departamento de Educação e Pesquisa e pela Comissão de Ética do Centro Hospitalar Universitário do Porto. Está em conformidade com a Declaração de Helsinque, com as diretrizes para a conduta ética de pesquisas médicas envolvendo crianças e com a legislação nacional vigente.

### Análise Estatística

A análise estatística padrão foi realizada com o software IBM SPSS Statistics for Macintosh, versão 28.0.1.0 (Armonk, NY: IBM Corp, EUA). As variáveis são apresentadas como mediana e percentis 25 e 75 ou n (%), conforme apropriado. As diferenças entre os grupos para variáveis contínuas foram avaliadas com o teste de Mann-Whitney. O teste qui-quadrado foi utilizado para comparação de proporções de variáveis categóricas. Um valor de p inferior a 0,05 foi considerado significativo.

## Resultados

Foram incluídos na análise 46 pacientes pediátricos com idade mediana (percentil 25–75, P25–75) de 13,0 (3,5–15,5) anos. As características demográficas dos parâmetros clínicos e analíticos são mostradas na Tabela 1, de acordo com os estágios KDIGO [estágio 1, 10 (21,7%); estágio 2, 12 (26,1%); estágio 3, 24 (52,2%)]. O principal mecanismo patogênico relatado foi o de causas renais intrínsecas (73,9%). As etiologias mais comuns de IRA foram nefrite intersticial aguda (23,9%), desidratação/choque (21,7%) e glomerulonefrite aguda (19,6%). Aproximadamente metade dos pacientes (n = 24; 52,2%) apresentou um fator de risco identificável para o desenvolvimento de IRA, sendo as comorbidades mais comuns (37,5%) as patologias (renais: 1 síndrome nefrótica e 1 hematúria/proteinúria em investigação; cardiovasculares: 1 hipertensão, 1 comunicação interventricular e 1 trombose da veia jugular interna; hemato-oncológicas: 1 osteoma osteoide; autoimune: 1 diabetes tipo 1 e lúpus eritematoso sistêmico e 1 hepatite autoimune), seguidas por exposição a nefrotoxinas (25,0%) e pela presença de CAKUT (25,0%). Com relação à classificação da IRA quanto ao débito urinário, 13 pacientes (28,3%) eram anúricos, 7 (15,2%) eram oligúricos e 26 (56,5%) eram não oligúricos. Todos os pacientes anúricos foram classificados como IRA KDIGO 3. Nove (19,5%) pacientes apresentaram sobrecarga hídrica e todos foram classificados como IRA estágio 3 (considerando valores de creatinina corrigidos para sobrecarga hídrica). A proporção de pacientes com hiponatremia [KDIGO estágio 1 *vs.* estágio 2 *vs.* estágio 3: 0 (0,0%) *vs.* 1 (8,3%) *vs.* 8 (33,3%), respectivamente, p = 0,043]; hipercalemia [KDIGO estágio 1 *vs.* estágio 2 *vs.* estágio 3: 0 (0,0%) *vs.* 1 (8,3%) *vs.* 8 (33,3%), respectivamente, p = 0,043] e acidose metabólica [KDIGO estágio 1 *vs.* estágio 2 *vs.* estágio 3: 1 (10,0%) *vs.* 0 (0,0%) *vs.* 10 (41,7%), respectivamente, p = 0,011] aumentou entre os estágios de IRA. A proporção de pacientes com hipertensão foi maior entre pacientes com IRA estágio 3, mas a diferença não foi estatisticamente significativa. Foi realizada biópsia renal em 10 pacientes, 5 dos quais apresentaram IRA estágio 3 (4 nefrite intersticial aguda). Quase um quarto (n = 10; 21,7%) dos pacientes necessitaram de terapia renal substitutiva, a saber, diálise peritoneal, hemodiálise ou ambas as técnicas, todos pertencentes ao grupo IRA estágio 3. Dez (21,7%) pacientes foram internados em uma unidade de terapia intensiva, 90% dos quais apresentavam IRA estágio 3.

**Tabela 1:** Parâmetros clínicos e analíticos de acordo com os estágios KDIGO de IRA

	Total 46	Estágio KDIGO			*p*
		1 10 (21,7%)	2 12 (26,1%)	3 24 (52,2%)	
**Dados demográficos e antropométricos**					
Idade (anos)	12,96 (3,54–15,54)	13,50 (9,17–15,52)	14,21 (7,15–16,98)	10,54 (1,40–15,08)	0,109
Sexo masculino	24 (52,2%)	3 (30,0%)	7 (58,3%)	14 (58,3%)	0,284
**Patogênese e diagnóstico da IRA**					
*Pré-renal*	9 (19,6%)	0 (0,0%)	2 (16,7%)	7 (29,2%)	0,122
Desidratação/choque	10 (21,7%)	1 (10,0%)	2 (16,7%)	7 (29,2%)	0,116
*Renal*	34 (73,9%)	8 (80,0%)	10 (83,3%)	16 (66,7%)	0,122
Nefrite intersticial aguda	11 (23,9%)	2 (20,0%)	3 (25,0%)	6 (25,0%)	0,116
Glomerulonefrite aguda	9 (19,6%)	2 (20,0%)	5 (41,7%)	2 (8,3%)	0,116
Síndrome hemolítico-urêmica	6 (13,0%)	0 (0,0%)	0 (0,0%)	6 (25,0%)	0,116
Nefrotoxicidade	4 (8,7%)	2 (20,0%)	1 (8,3%)	1 (4,2%)	0,116
Síndrome nefrótica	1 (2,2%)	0 (0,0%)	0 (0,0%)	1 (4,2%)	0,116
*Pós-renal*	3 (6,5%)	2 (20%)	0 (0,0%)	1 (4,2%)	0,122
Obstrução	3 (6,5%)	2 (20,0%)	0 (0,0%)	1 (4,2%)	0,116
Desconhecido	2 (4,3%)	1 (10,0%)	1 (8,3%)	0 (0,0%)	0,116
**Fator de risco**	24 (52,2%)	6 (60,0%)	7 (58,3%)	11 (45,8%)	0,665
Comorbidades	9 (37,5%)	1 (16,7%)	3 (42,9%)	5 (45,5%)	0,409
Nefrotóxico	6 (25,0%)	2 (33,3%)	3 (42,9%)	1 (9,1%)	0,409
Prematuridade	1 (4,2%)	1 (16,7%)	0 (0,0%)	0 (0,0%)	0,409
CAKUT	6 (25,0%)	1 (16,7%)	1 (14,3%)	4 (36,4%)	0,409
Nefrolitíase	2 (8,3%)	1 (16,7%)	0 (0,0%)	1 (9,1%)	0,409
**Débito urinário**					
Anúria	13 (28,3%)	0 (0,0%)	0 (0,0%)	13 (54,2%)	<**0,001**
Oligúria	7 (15,2%)	1 (10,0%)	5 (41,7%)	1 (4,2%)	**<0,001**
Não oligúria	26 (56,5%)	9 (90,0%)	7 (58,3%)	10 (41,7%)	**<0,001**
**Hipertensão**	17 (37,0%)	3 (30,0%)	3 (25,0%)	11 (45,8%)	0,416
**Parâmetros bioquímicos**					
Hiponatremia	9 (19,6%)	0 (0,0%)	1 (8,3%)	8 (33,3%)	**0,043**
Hipercalemia	9 (19,6%)	0 (0,0%)	1 (8,3%)	8 (33,3%)	**0,043**
Acidose metabólica	11 (23,9%)	1 (10,0%)	0 (0,0%)	10 (41,7%)	**0,011**
Proteinúria	29 (63,0%)	6 (60,0%)	7 (58,3%)	16 (66,7%)	0,865
Hematúria	20 (43,5%)	6 (60,0%)	5 (41,7%)	9 (37,5%)	0,478
**Biópsia renal**	10 (21,7%)	1 (10,0%)	4 (33,3%)	5 (20,8%)	0,413
**Terapia renal substitutiva**	10 (21,7%)	0 (0,0%)	0 (0,0%)	10 (41,7%)	**0,003**
Hemodiálise	2 (4,3%)	0 (0,0%)	0 (0,0%)	2 (8,3%)	
Diálise peritoneal	6 (13,0%)	0 (0,0%)	0 (0,0%)	6 (25,0%)	
Ambos	2 (4,3%)	0 (0,0%)	0 (0,0%)	2 (8,3%)	
**Necessidade de unidade de terapia intensiva**	10 (21,7%)	1 (10,0%)	0 (0,0%)	9 (37,5%)	**0,022**
Ventilação mecânica	3 (6,5%)	0 (0,0%)	0 (0,0%)	3 (6,5%)	0,230
Ionotrópicos	2 (4,3%)	0 (0,0%)	0 (0,0%)	2 (8,3%)	0,384

IRA – Insuficiência renal aguda; CAKUT – Anomalias congênitas do rim e do trato urinário.

Os valores são relatados como mediana (percentil 25-percentil 75) ou n (%).

A caracterização das sequelas de acordo com os estágios da IRA é mostrada na Tabela 2. Dos 46 pacientes originais, 2 foram perdidos durante o seguimento e, consequentemente, não foram incluídos na análise. Não houve óbitos. A maioria dos pacientes (n = 26; 59,1%) apresentou pelo menos uma sequela de 3 a 6 meses após a alta. A frequência de sequelas aumentou entre os estágios KDIGO de IRA. As sequelas mais frequentes foram proteinúria (n = 15, 38,5%; mediana (P25–75) da PU/CU 0,30 (0,27–0,44) mg/mg), seguida por TFG reduzida (n = 11, 27,5%; mediana (P25–75) da TFG 75 (62–83) mL/min/1,73 m^2^) e hipertensão (n = 4, 9,1%). Entre os pacientes sem sequelas no acompanhamento, os valores medianos da PU/CU e da TFG foram 0,10 (0,06–0,15) mg/mg e 108 (100–119) mL/min/1,73 m^2^, respectivamente. Embora dentro da faixa normal, a mediana da TFG no acompanhamento aumentou entre os estágios da IRA [KDIGO estágio 1 *vs.* estágio 2 *vs.* estágio 3: 93 (93-93) *vs.* 107 (103–115) *vs.* 115 (106–137) mL/min/1,73 m^2^, respectivamente, p = 0,035]. Doze dos 15 pacientes com proteinúria iniciaram terapia com inibidor da enzima conversora da angiotensina ou bloqueador do receptor da angiotensina II durante o acompanhamento.

**Tabela 2:** Caracterização da sequela de acordo com os estágios KDIGO de IRA

	Total n = 44^a^	Estágio KDIGO			*p*
		1	2	3	
**Sem qualquer sequela**	**18 (40,9%)**	**6 (60,0%)**	**6 (54,5%)**	**6 (26,1%)**	0,108
PU/CU (mg/mg)	0,10 (0,06–0,15)	0,09 (0,06–0,15)	0,11 (0,07–0,16)	0,11 (0,06–0,15)	0,778
TFG (mL/min/1,73 m^2^)	108 (100–119)	93 (93-93)	107 (103–115)	115 (106–137)	**0,035**
**Com sequela**	**26 (59,1%)**	**4 (40,0%)**	**5 (45,5%)**	**17 (73,9%)**	0,108
**Proteinúria** ^b^	15 (38,5%)	2 (22,2%)	2 (18,2%)	11 (57,9%)	0,051
PU/CU (mg/mg)	0,30 (0,27–0,44)	(0,29–0,36)^c^	(0,23–0,29)^c^	0,30 (0,27–0,47)	0,792
**TFG reduzida** ^d^	11 (27,5%)	2 (28,6%)	2 (18,2%)	7 (31,8%)	0,709
TFG (mL/min/1,73 m^2^)	75 (62–83)	(71–77)^c^	(75–79)^c^	72 (59–80)	0,633
**Hipertens**ã**o** ^e^	4 (9,1%)	0 (0,0%)	2 (18,2%)	2 (8,7%)	0,349

TFG – Taxa de filtração glomerular; KDIGO – Kidney Disease Improving Global Outcomes; PU/CU – Relação proteína/creatinina urinária.

Os valores são relatados como n (%) e mediana (percentil 25–75).

a Dos 46 pacientes iniciais, 2 foram perdidos no acompanhamento e, consequentemente, não foram incluídos na análise.

b Proteinúria foi definida como uma relação de proteína/creatinina urinária >0,2 mg/mg.

c Devido ao baixo número de pacientes nessa categoria (menos de 3), os valores medianos (percentis 25–75) não puderam ser calculados e os valores reais de cada paciente são apresentados.

d TFG reduzida foi definida como TFG < 90 mL/min/1,73 m^2^.

e Hipertensão foi definida como valores sistólicos ou diastólicos acima do percentil 95 para idade, sexo e altura^25^.

## Discussão

No presente estudo, relatamos a etiologia, gravidade e desfechos da IRA entre pacientes internados em uma Unidade de Nefrologia Pediátrica de um hospital terciário durante a última década.

A maioria dos casos de IRA foram associados a causas renais intrínsecas, especialmente nefrite intersticial aguda, glomerulonefrite aguda e síndrome hemolítico-urêmica, seguidas por causas pré-renais, nomeadamente desidratação/choque. Embora diversos estudos relatem que a IRA pediátrica é derivada principalmente de etiologias pré-renais^14,17,18^, a predominância de causas renais pode estar relacionada à natureza altamente diferenciada de nosso centro. Por sermos o centro de referência em pacientes pediátricos com doenças renais para toda a região norte do país, a proporção de etiologias renais pode estar sobrerrepresentada em nossa amostra.

Sabe-se que pacientes com comorbidades são altamente suscetíveis à IRA^1,2,5^. A síndrome nefrótica, por exemplo, é uma causa frequente de doença renal em crianças e a IRA é descrita como uma complicação potencial^26,27^. Embora a incidência de IRA em crianças com síndrome nefrótica seja variável entre os estudos, um estudo encontrou IRA em cerca de metade de sua população^27^. No entanto, em nosso estudo, apenas um paciente apresentou IRA com uma recidiva de síndrome nefrótica. As doenças cardiovasculares, como insuficiência cardíaca e cardiopatias congênitas, também impõem um risco significativo de IRA^28^. A IRA é particularmente comum em crianças submetidas a cirurgia cardíaca, com estudos sugerindo uma correlação significativa entre formas moderadas a graves de lesão e mortalidade pós-operatória^29^, o que está em conformidade com nossos achados. A IRA é uma comorbidade comum das doenças hemato-oncológicas e relatos indicam que, nesses pacientes, a IRA nos estágios 2 e 3 está associada a uma maior mortalidade^30^. As doenças autoimunes também podem levar ao desenvolvimento de IRA, com potencial de progredir rapidamente para formas graves de lesão^31^. Em nossa coorte, cerca de um terço dos pacientes apresentou um histórico prévio de doenças renais, cardiovasculares, hemato-oncológicas ou autoimunes. Também é importante observar que a maioria desses pacientes desenvolveu IRA moderada a grave, correspondendo aos estágios do KDIGO 2 e 3, portanto, consistente com relatos anteriores.

A oligoanúria foi relatada em aproximadamente metade dos pacientes internados, onde quase todos desenvolveram IRA nos estágios 2 e 3, sugerindo que essas alterações no débito urinário podem representar um risco para formas mais graves da doença e, portanto, desfechos potencialmente piores, conforme relatado anteriormente na literatura^32^.

Os pacientes com casos mais graves de IRA foram aqueles com mais distúrbios nos parâmetros bioquímicos, como hiponatremia, hipercalemia e acidose metabólica. Esses achados sugerem que insultos renais graves estão associados a distúrbios hidroeletrolíticos mais pronunciados e são consistentes com estudos que sugerem uma associação entre anormalidades eletrolíticas, principalmente acidose metabólica, e pior prognóstico em crianças com IRA^33,34^.

Relatamos que todos os pacientes que necessitaram de terapia renal substitutiva foram categorizados no estágio mais grave de IRA, o que está de acordo com estudos anteriores^18,35^. A diálise peritoneal foi o método de terapia renal substitutiva mais comumente utilizado, o que é consistente com diversos estudos que relatam que a diálise peritoneal é um método bem tolerado, fácil de executar e com eficácia conhecida no contexto da IRA pediátrica^36–38^. Além disso, a hemodiálise requer um bom funcionamento do acesso vascular e pacientes hemodinamicamente estáveis, sendo, portanto, reservada para ambientes mais específicos^38^. Embora as terapias renais substitutivas contínuas tendam a ser a modalidade de escolha em pacientes críticos e hemodinamicamente instáveis^39,40^, a diálise peritoneal foi a terapia renal substitutiva mais comumente utilizada para os pacientes na UTIP, e nenhuma terapia contínua foi usada em nossa população durante o período do estudo.

Em nosso estudo, constatamos que a maioria dos pacientes que necessitaram de terapia renal substitutiva também foi admitida na UTIP durante o curso da internação, destacando a gravidade inerente à IRA estágio 3. Embora estudos anteriores tenham encontrado uma correlação entre a gravidade da IRA e a necessidade e duração da ventilação mecânica^16,41^, não encontramos uma diferença estatisticamente significativa na necessidade de ventilação mecânica em diferentes estágios da IRA. Isso pode ser devido ao pequeno número de pacientes em nossa população que necessitaram de tratamento na UTIP e ventilação mecânica. As baixas taxas de utilização de ventilação mecânica e drogas vasoativas em nossa coorte de estudo podem sugerir uma menor gravidade dos casos em comparação com outras séries e podem contribuir para a ausência de óbitos em nossa coorte.

Embora não tenha havido óbitos em nosso estudo, destacamos o fato de que quase 60% dos pacientes apresentaram pelo menos uma sequela de 3 a 6 meses após a alta hospitalar, com mais de 25% apresentando TFG reduzida no acompanhamento, portanto, sem recuperar completamente a função renal normal. O achado de valores medianos da TFG que aumentam na consulta de acompanhamento entre os estágios de IRA, com valores mais elevados em pacientes com IRA mais grave, parece contraintuitivo, mas pode representar um estágio inicial de hiperfiltração em pacientes com perda mais grave de néfrons durante o episódio de IRA, como relatado anteriormente na literatura^42–44^.

Reconhecemos que nosso estudo teve algumas limitações, particularmente o desenho retrospectivo e a experiência de um único centro de atendimento terciário. Apesar dessas limitações, acreditamos ter descrito uma população bastante representativa de pacientes pediátricos com IRA da região norte de nosso país durante um longo período de tempo. Consideramos que o estudo apresentado contribui para aumentar o conhecimento sobre a epidemiologia da IRA, uma área que necessita de mais estudos para promover a conscientização sobre as consequências de longo prazo da IRA na pediatria.

Em conclusão, a IRA foi comum no ambiente pediátrico, principalmente em pacientes com comorbidades prévias, mas também afetou crianças sem um fator de risco conhecido, enfatizando a importância da suspeita precoce dessa condição. Também descobrimos que a maior gravidade da IRA esteve associada a distúrbios eletrolíticos, à necessidade de terapias renais substitutivas e à admissão na UTIP. Nossos resultados sugerem que a IRA pode estar associada a uma morbidade significativa, especialmente ao desenvolvimento de proteinúria e à redução da TFG e, portanto, ao comprometimento da função renal. Isso destaca a necessidade de estudos adicionais com foco no impacto de longo prazo da IRA a fim de compreender melhor o potencial de consequências transitórias ou permanentes, com impacto importante no acompanhamento e manejo de longo prazo desses pacientes.

##  

Este documento possui uma errata: https://doi.org/10.1590/2175-8239-JBN-2024-0012erpt
